# Depression with Panic Episodes and Coronary Vasospasm

**DOI:** 10.1155/2009/453786

**Published:** 2009-06-01

**Authors:** Mladen I. Vidovich, Aneet Ahluwalia, Radmila Manev

**Affiliations:** ^1^Division of Cardiology, Department of Medicine, University of Illinois at Chicago, 840 South Wood Street, MC 715, Suite 935, Chicago, IL 60612, USA; ^2^Heart and Mind Clinic, University of Illinois at Chicago, Chicago, IL 60612, USA; ^3^Department of Psychiatry, University of Illinois at Chicago, Chicago, IL 60612, USA

## Abstract

Variant (Prinzmetal's) angina is an uncommon cause of precordial pain caused by coronary vasospasm and characterized by transient ST elevation and negative markers of myocardial necrosis. This is the case of a female patient with a prior history of depression and panic attacks who presented with recurrent symptoms including chest pain. A cardiac event monitor positively documented coronary vasospasm associated with anxiety-provoking chest pain, whereas the coronary arteries were angiographically normal. We noted that the frequency of angina attacks apparently increased during the period that coincided with the introduction of Bupropion SR for treatment of the patient's depression. Considering the possibility of bupropion-associated negative impact on coronary vasospasm, the antidepressant therapy was adjusted to exclude this drug. Although Prinzmetal's angina is relatively uncommon, we suspect that a routine use of cardiac event monitors in subjects with panic disorder might reveal a greater incidence of coronary vasospasm in this patient population.

## 1. Introduction

Almost one third of patients with chest pain who undergo coronary angiography have no apparent arterial abnormalities [1]. Some cases of such chest pain are diagnosed as Prinzmetal's, variant angina [2], in which pain typically occurs at rest and is caused by intense coronary vasospasm as opposed to typical angina, which is provoked by exercise and is most commonly due to coronary atherosclerosis. Frequently, when no coronary artery abnormalities are found, chest pain may be associated and is often attributed to panic disorder [3]. A history of the comorbidity of psychiatric disorders in patients who present with chest pain further complicates diagnostic and therapeutic decision-making. Here we report the case of a patient with a history of depression and anxiety who presented to the emergency room with chest pain.

## 2. Case Report

This is the case of 61-year-old African-American woman with a history of depression and anxiety who presented to the emergency room in May 2008 with an acute episode of left-sided chest pain/pressure with associated shortness of breath, diaphoresis, and an impending sense of doom lasting 2-3 minutes. There was no identifiable precipitant to this event.

She was admitted and placed on a cardiac telemetry monitoring. In the hospital, she was free of chest pain. The electrocardiogram, telemetry, and serial cardiac enzymes did not reveal myocardial infarction or any acute cardiac event. There was no family history of coronary artery disease. Medical history included hypertension, obesity, sleep apnea, and a history of smoking (quit 10 years prior) and alcohol abuse (from age 40 to 51). During hospitalization, the patient voiced concern that her chest pain may have been related to a recent recurrence of depressive symptoms. She recalled having similar chest pain 10 years ago. At that time, she had had cardiac testing including angiography, which was normal, and the pain was diagnosed as panic attacks. At that time (10 years ago) some relief was provided by diazepam and sertraline. As a young adult, the patient described low-level depression with suicidal ideation. Her other past psychiatric medications included fluoxetine, buspirone, trazodone, and risperidone. Her family psychiatric history included a father with depression, a brother with alcohol abuse, and one son with depression, alcohol abuse, and completed suicide (7 years ago). At the time of hospitalization, the patient was divorced with one living son. At discharge, she was scheduled for an outpatient stress test.

Six days after hospitalization, she was seen in the medical clinic. She was free of chest pain but reported depression with decreased energy, lethargy, and decreased interest in activities. Therapy was started with 25 mg of sertraline for 2 weeks, which was titrated up to 50 mg at week 3. A referral was made to the psychiatry department.

One month after being started on sertraline (early June), she was seen in psychiatry clinic. At that time, she was tolerating 50 mg of sertraline but without much benefit. She described depression with anergia and a lower level of anxiety than she had previously in psychiatric care years ago. She also reported 2 episodes of chest pain with tremor and sensation of heart racing in the past month. Laboratory analyses were ordered to rule out organic fatigue, and a referral was made to a sleep clinic as she had been diagnosed with sleep apnea 8 years before by a polysomnogram (PSG) test, but failed to follow up. Bupropion (Bupropion SR, 100 mg) was added to sertraline to address her depression with anergia, and alprazolam was given for anxiety as needed. Two and a half weeks after being started on bupropion the patient was still complaining of depression and fatigue, and bupropion was increased to 150 mg.

Three weeks after the bupropion dose change, the PSG results came back revealing sleep apnea, and the patient was placed on continuous positive airway pressure (CPAP) therapy. A week after PSG results were received, during her sleep clinic appointment, the patient experienced substernal chest pain with pressure radiating down her left arm. The physician from the sleep clinic made a referral to cardiology, as she had failed a previous appointment for cardiology and a stress test. In a psychiatric follow-up (one week after her sleep clinic appointment), she was feeling much better, including improved mood and less fatigue and anxiety.

**Figure 1:**
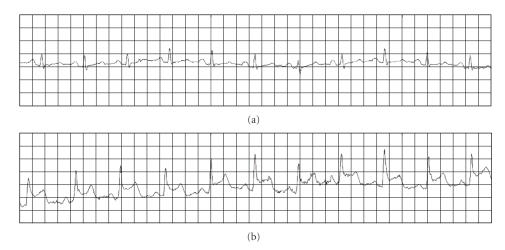
*Representative ECG recordings*. Panel (a) shows the baseline ECG. Panel (b) shows ECG recordings during the chest pain and reveals the ST segment elevations.

One week later, the patient was seen in the cardiology outpatient clinic describing a 3-month history of frequent (10 or 15) episodes of severe midsternal chest pain lasting 5 to 10 minutes that was associated with left arm weakness. The symptoms were neither exercise-induced nor predictable. The symptoms were extremely anxiety-provoking but she reported no associated diaphoresis or shortness of breath. The electrocardiogram revealed sinus rhythm and left ventricular hypertrophy. The cholesterol panel was unremarkable. Due to worrisome clinical presentation, coronary angiography was performed. The coronary arteries were angiographically normal. There were no wall motion abnormalities, and the ejection fraction was normal. The patient was discharged with a cardiac event monitor. Within 5 days the patient experienced two recurrences of her symptoms. The event monitor revealed ST elevations that were associated with anxiety-provoking chest pain (Figure 1). There were no significant changes in heart rate and no arrhythmias were recorded. The patient was diagnosed with vasospastic (Printzmetal's or variant) angina and started on amlodipine (2.5 mg daily) and isosorbide mononitrate (30 mg daily). 

In a subsequent psychiatric follow-up, the patient was free of chest pain and panic attacks. Bupropion was tapered off, and sertraline was increased to 75 mg daily because it was feared that bupropion could contribute to or worsen her coronary vasospasm.

## 3. Discussion

Prinzmetal's-variant angina is an uncommon cause of precordial pains, characterized by transient ST elevation and negative markers of myocardial necrosis [2, 4]. This type of angina is caused by an intense coronary vasospasm of unclear etiology. A definitive diagnosis requires demonstration of spontaneous or test-provoked (e.g., by hyperventilation, acetylcholine, or ergonovine) ST-segment elevation. Based on data from test-provoked vasospasms, the reported incidence of variant angina ranges from 4% to 32% [5]. In our patient, the spontaneous ST-segment elevation associated with the anxiety-provoking chest pain was documented using a cardiac event monitor.

There are no studies on the incidence of panic disorder in patients with Prinzmetal's angina/chest pain, but about one third of chest pain patients with angiographically normal coronary arteries reportedly have panic disorder [1, 6]. It is worth noting that hyperventilation, a proposed provocative test for diagnosing Prinzmetal's angina [7], typically accompanies panic attacks and hence could trigger coronary vasospasm in susceptible subjects. Although no systematic research has been conducted on the occurrence of coronary vasospasm in patients with panic disorder, several cases of coronary vasospasm leading to ischemia were documented in these patients [8]. These latter cases appear to be less common in patients with panic disorder and bear similarity to conditions of acute stress cardiomyopathy, also known as Takotsubo cardiomyopathy or broken heart syndrome [9].

The case we present here is unusual because in addition to documented Prinzmetal's angina, the main psychiatric component of the disorder presented as depression with panic attacks rather than typical panic disorder. Furthermore, although the patient had been diagnosed with panic attacks 10 years prior, chest pain was mostly absent until the episode that led to her most recent hospitalization. Moreover, the frequency of angina attacks increased dramatically (up to 15 episodes in 3 months) during the period that coincided with the introduction of bupropion for treatment of the patient's depression.

It has been suspected that bupropion may be associated with chest pain [10] and myocardial infarction. In two cases, young males were diagnosed with ST elevations, normal coronary angiogram, and myocardial infarction, which was associated with the use of bupropion [11, 12]. The main mechanism of bupropion's action is believed to be the inhibition of the central nervous system presynaptic dopamine and norepinephrine reuptake transporters. In addition, it appears that bupropion exerts a direct action on the human myocardium, presumably by triggering catecholamine release [13]. Since our patient had a diagnosis of prior angina attacks, it is possible that her recent angina could have been exacerbated by treatment with bupropion. While majority of patients with Prinzmetal's angina stabilize and improve after the initial 3–6 month period of frequent symptoms, recrudescence has been observed. The triggers for the return of symptoms have not been elucidated [14]. 

## 4. Conclusion

This patient, who had a prior history of depression and panic attacks, presented with recurrent symptoms including chest pain. Using a cardiac event monitor, she was documented to be suffering from coronary vasospasm that was associated with anxiety-provoking chest pain. We noted that the frequency of angina attacks apparently increased during the period that coincided with the introduction of bupropion for treatment of the patient's depression. Considering the possibility that bupropion may have a negative impact on coronary vasospasm, the antidepressant therapy was adjusted to exclude this drug. Although Prinzmetal's angina is relatively uncommon, further studies are needed to assess the routine use of cardiac event monitor in subjects with panic disorder. Furthermore, this case is a good example of how a close collaboration between cardiologists and psychiatrists can improve patient care.
